# The reversible effects of gossypol toxicity on male pigeons’ reproductive performance

**DOI:** 10.14202/vetworld.2022.2836-2843

**Published:** 2022-12-13

**Authors:** Suwarak Wannaratana, Wijit Banlunara, Kaj Chokeshaiusaha, Thanida Sananmuang

**Affiliations:** 1 Faculty of Veterinary Medicine, Rajamangala University of Technology Tawan-Ok, Chonburi 20110, Thailand; 2 Department of Pathology, Faculty of Veterinary Science, Chulalongkorn University, Bangkok 10330, Thailand

**Keywords:** gossypol, pigeon, sperm morphology, sperm motility, sperm viability

## Abstract

**Background and Aim:**

Gossypol, a cotton seed derivative, is well known for its reversible antifertility in male reproduction across species. Its antifertility and reversibility effects on male reproductive function vary among species in dose-and time-dependent manners. In this study, the antifertility potential of gossypol in pigeons was evaluated for the first time to determine whether it might be used as a dietary supplement for pigeon population control.

**Materials and Methods:**

Male pigeons were assigned into three experimental groups: The gossypol-treated group (n = 12), the sham control group (n = 6), and the negative control group (n = 6). There were two experimental periods: A gossypol-feeding period of 28 days and a gossypol-free period of 28 days. During the gossypol-feeding period, birds in the gossypol-treated group were fed 4 mg of gossypol extract per day. Birds in the sham control group were fed 0.5 mL of mixed ethanol and sunflower oil, while those in the negative control group were fed 0.5 mL of phosphate buffer saline. After the gossypol-feeding phase was completed, all remaining pigeons in all groups continued to receive their regular diet for an additional 28 days (gossypol-free phase). The body weight and semen quality of the birds in the experimental groups were compared to evaluate gossypol’s antifertility effect.

**Results:**

In the gossypol-treated group as compared to the control groups, the percentages of sperm motility and viability were significantly lower at 21 days, and the percentage of normal sperm morphology was significantly lower at 28 days during the gossypol-feeding period. After gossypol withdrawal, these antifertility effects were resumed and reached a comparable semen quality to the control groups within 14 days.

**Conclusion:**

Gossypol supplementation (4 mg/day for 28 days) could lower male pigeons’ reproductive performance in terms of sperm motility, viability, and sperm morphology. Such infertility was, however, reversible within 14 days after gossypol withdrawal without any side effects on the pigeons, suggesting its application as a safe contraceptive feeding for male pigeons.

## Introduction

In traditional Chinese medicine, gossypol, a natural polyphenolic compound derived from cotton seeds, is recognized as one of many testicular toxicants [1–3]. Besides the infertile effect on male reproduction, other effects such as anti-parasite, anti-inflammatory, and anti-tumor properties are also reported [4–8]. Based on several previous studies, the toxic effects of gossypol on male reproductive performance have varied among species [9], such as enhancing sperm abnormalities in bulls [10], reducing sperm count and motility in rats [11], and increasing spermatocyte degeneration in hamsters [12]. Such toxicities also show dose-dependent, time-dependent, and even reversible characteristics [10, 13, 14]. Studies in avian species also supported the unique reversible characteristics of gossypol toxicity in a species-specific manner. For example, domestic cocks fed with an effective dose of gossypol at 40 mg/kg body weight (BW) per day for 18 days had a full recovery of semen quality within 4 weeks after cessation of gossypol treatment [15]. In Japanese quail, the supplement gossypol at a dose of 25 mg/kg BW per day for 12 and 24 days resulted in severe infertility of the birds by which their complete recovery could be acquired after 11 and 20 days of gossypol withdrawal, respectively [14]. Due to the species-dependent characteristics of gossypol, its dose-dependent and time-dependent effects require separate investigation whenever applied to a novel bird species.

Feral pigeons (*Columba livia domestica*) are common pests worldwide, especially in almost every large city [16]. The overpopulated pigeons are causes of several consequential problems such as stress, parasites, and diseases relating to the poor living condition of humans [17, 18]. Moreover, pigeon droppings also cause a huge economic impact due to their infrastructure deterioration [19]. By means of this, a variety of efforts were enforced with the major aim of controlling and reducing the pigeon population, including trapping, poisoning, ultrasonic repelling, and culling. However, these procedures raise contentious questions about animal welfare and the unpredictable effects on non-target species [20]. Even though the non-invasive hormonal treatment for pigeon population control was previously suggested, the stabilization of the program was still not practical and had become the major limitation of the method [21]. Therefore, a better strategy for pigeon population control is required. From this, fertility control by reducing the birth rate is thus a suitable method for pigeon population control in terms of morality and social acceptance [22].

This study aimed to evaluate the non-invasive infertile effect of gossypol on male pigeons’ reproduction. For the first time, this study reported the antifertility effect and reversibility of gossypol on male pigeons. With the determined dose and timing of gossypol treatment utilized, the results acquired from this study would provide fundamental data for the gossypol-based contraceptive drug for pigeons’ population control in the future.

## Materials and Methods

### Ethical approval

Experimental procedures were approved by the Rajamangala University of Technology Tawan-OK Animal Ethics Committee (RMUTTO-ACUC-2-2019-003), and care was taken to minimize the number of animals used.

### Study period and location

The study was conducted from August to September 2019 at the Faculty of Veterinary Medicine, Rajamangala University of Technology Tawan-OK in Chonburi province, Eastern part of Thailand.

### Experimental design

A total of 24 fertile male racing pigeons, aged between 1 and 2 years, were used in this study. All birds were Belgian-bred and contributed by the racing pigeon international association (Thailand). They were kept individually in metal cages and placed in the open house in their natural environment. They were fed 60 g of commercial bird feed once a day and had access to water available *ad libitum*. In this study, they were randomly divided into three experimental groups: A gossypol-treated group (n = 12), a sham control group (n = 6), and a negative control group (n = 6). The gossypol-treated group was additionally fed with 0.5 mL of gossypol extract (Sigma-Aldrich, St. Louis, USA) dissolved in ethanol (Merck KGaA, Darmstadt, Germany), then diluted in sunflower oil to give a gossypol concentration of 4 mg/0.5 mL. The birds of the sham control group were fed 0.5 mL of 10% ethanol diluted in 0.45 mL of sunflower oil. For the negative control group, the birds were fed with 0.5 mL of phosphate buffer saline.

**Figure-1:**
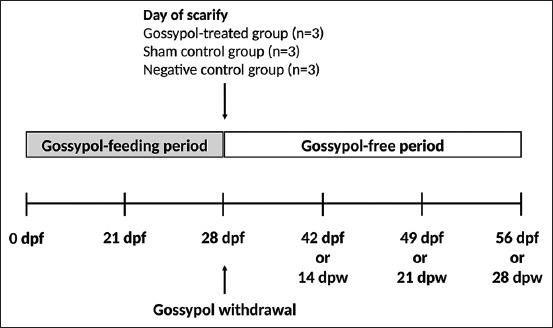
Diagram of experimental design. DPF=Days post-gossypol feeding, DPW=Days post-gossypol withdrawal.

This study consisted of two experimental periods; the gossypol-feeding period (28 days) and the gossypol-free period (28 days). The gossypol-feeding period aimed to determine the effect of gossypol on spermatogenesis and reproductive performance in male pigeons. Their BW and semen quality were evaluated at day 0 (the day before the gossypol treatment), 21, and 28 of the gossypol-feeding period, respectively. On day 29, the three candidate pigeons from each group were sacrificed for testicular and blood sample collection (Figure-1). Testicular histopathology among the experimental groups was examined to evaluate the effect of gossypol on germ cell development. Hematology and serum biochemistry among the experimental groups were assessed to determine the toxicity of gossypol.

The gossypol-free period started after finishing the first one to determine the reversible effect of gossypol on the reproductive performance of male pigeons. During this period, the remaining pigeons in each group – gossypol-treated group (n = 9), sham control group (n = 3), and negative control group (n = 3) were maintained on only a normal diet for 28 days without any supplement. The BW and semen quality of the birds were evaluated on day 0 (the last day of gossypol treatment), 14, 21, and 28 of the gossypol-free period, respectively (Figure-1).

### Body weight, semen collection, and semen evaluation

The BW and semen quality of each pigeon were evaluated before feeding time in the morning on the days as described in the experiment design. The semen was collected by lumbosacral massaging and gentle squeezing at the base of the cloaca, performed by the same collector. The obtained semen was 25-fold diluted in Lactated ringer’s solution (General Hospital Products Public Co. Ltd., Thailand) before being promptly assessed for quality [23]. The macroscopic evaluations included semen volume, pH, and color determination. The microscopic evaluations included total sperm motility, progressive sperm motility, sperm viability, sperm concentration, total sperm count, and sperm morphological analysis.

A calibrated displacement pipette was used to measure the volume of the ejaculates, and pH indicator strips (Riedel-De-Haёn AG, Victoria, Australia) were used to assess the pH. For standardization, each of the evaluation techniques was performed by the same evaluator. The percentage of total motile sperm and progressive motility were evaluated under the light microscope at 100× magnification for at least 10 fields. Progressive motility of the sperm was graded on a 5-point scale (where 0 indicates no motility, 1 indicates non-progressive motility, 2 indicates slow progressive motility, 3 indicates side-to-side movement accompanied by slow progressive motility, 4 indicates faster progressive motility, and 5 indicates very fast progressive motility) [24].

Semen samples stained with eosin-nigrosin dye (Pornchai Intertrade Co., Ltd., Thailand) were assessed for sperm viability and morphology. Based on 200 sperm counts, the percentages of living (eosin-impermeable) and dead (eosin-permeable) sperm in a sample were calculated. For morphology evaluation, 300 sperm counts acquired from each sample were assessed for normal, amorphous head, bent head, macrocephalic head, acrosomal defect, loosed head, abnormal midpiece, proximal droplet, coiled tail, bent tail, distal droplet, loosed tail, and double tail sperm under a light microscope (Nikon, Japan) at 1000× magnification.

Sperm concentration was assessed by diluting 10 μL of sperm suspension in 990 μL of formal saline (100-fold dilution). The diluted sperm suspension was transferred to a counting chamber (Boeco, Hamburg, Germany) and the sperm concentration was then evaluated under a light microscope at 400× magnification.

### Hematology, biochemistry, and histopathology

A total of approximately 1 mL of blood sample from either wing vein or cephalic vein were collected into both ethylenediamine tetraacetic acid (EDTA)-containing tubes and plain tubes. Blood samples collected in an EDTA-containing tube (Becton, Dickinson and Company, Plymouth, UK) were used for complete blood count evaluation. For biochemical analysis, serum obtained from blood samples collected in a simple tube was employed. Serum biochemistry included alanine aminotransferase, aspartate aminotransferase, alkaline phosphatase, bile acid, blood urea nitrogen, gamma-glutamyl transferase, lactate dehydrogenase, total protein, triiodothyronine, testosterone, and uric acid. For evaluation of testes’ histopathology, the collected testes were fixed in 10% buffered formalin, embedded in paraffin, sectioned at 4 mm, and then stained with hematoxylin and eosin. Under a light microscope (Carl Zeiss Co Ltd., Germany) at 40×, 100× and 400× magnification, the histopathological characteristics of testes and seminiferous tubules were compared among the experimental groups.

### Statistical analysis

All experimental groups’ data had a normal distribution with approximately equal variance. One-way analysis of variance with Tukey’s test for *post hoc* analysis was used for comparison among the experimental groups. Statistical significance was defined as p < 0.05.

## Results

### The effect of gossypol on spermatogenesis and reproductive performance in pigeons during the gossypol-feeding period

There were no significant differences in BW, semen volume, semen pH, semen concentration acquired among birds in all experimental groups during the gossypol-feeding period (Table-1). However, male pigeons in the gossypol-treated group demonstrated decreased sperm motility and viability on day 21 compared to day 0 of treatment. Such decreases were also significantly lower when compared to those acquired from all other control groups. In addition, the percentage of normal sperm morphology in the gossypol-treated group also decreased on day 28 of treatment when compared to those of other control groups (Table-1), in which a loose tail or sperm without a tail was the major sperm defect (Table-2).

**Table-1:** Body weight and semen quality of male pigeons were compared among gossypol-treated (n = 12), sham-control (n=6), and negative-control (n=6) groups at day 0, 21, and 28 of treatment.

Parameter	Gossypol-treated group (n = 12)	Sham-control group (n = 6)	Negative-control group (n = 6)
Body weight (g)			
Day 0	435.3 ± 5.20^a,A^	441.0 ± 13.55^a,A^	454.4 ± 7.05^a,A^
Day 21	424.7 ± 11.50^a,A^	452.9 ± 13.69^a,A^	453.1 ± 8.60^a,A^
Day 28	423.1 ± 12.86^a,A^	439.7 ± 7.15^a,A^	444.7 ± 8.56^a,A^
Semen volume (μL)			
Day 0	12.08 ± 1.56^a,A^	12.50 ± 1.71^a,A^	13.33 ± 2.11^a,A^
Day 21	8.83 ± 1.03^a,A^	10.50 ± 2.55^a,A^	10.83 ± 2.01^a,A^
Day 28	10.00 ± 0.87^a,A^	13.33 ± 2.79^a,A^	11.67 ± 2.11^a,A^
Semen pH			
Day 0	7.50 ± 0.11^a,A^	7.60 ± 0.05^a,A^	7.50 ± 0.04^a,A^
Day 21	7.62 ± 0.06^a,A^	7.47 ± 0.04^a,A^	7.50 ± 0.04^a,A^
Day 28	7.35 ± 0.09^a,A^	7.43 ± 0.11^a,A^	7.53 ± 0.07^a,A^
Total motility (%)			
Day 0	83.33 ± 2.45^a,A^	82.50 ± 4.43^a,A^	87.50 ± 4.43^a,A^
Day 21	34.17 ± 9.81^b,B^	80.00 ± 5.16^a,A^	85.00 ± 4.28^a,A^
Day 28	30.00 ± 9.94^b,B^	77.50 ± 6.80^a,A^	85.83 ± 2.71^a,A^
Progressive motility (score 0–5)			
Day 0	4.75 ± 0.13^a,A^	4.50 ± 0.22^a,A^	4.83 ± 0.17^a,A^
Day 21	2.50 ± 0.50^b,B^	4.33 ± 0.33^a,A^	4.50 ± 0.34^a,A^
Day 28	2.27 ± 0.52^b,B^	4.58 ± 0.27^a,A^	4.83 ± 0.17^a,A^
Viability (%)			
Day 0	86.38 ± 1.43^a,A^	85.17 ± 1.73^a,A^	83.00 ± 3.34^a,A^
Day 21	66.42 ± 2.92^b,B^	86.08 ± 2.26^a,A^	88.00 ± 2.13^a,A^
Day 28	67.24 ± 3.01^b,B^	83.92 ± 4.63^a,A^	86.42 ± 3.32^a,A^
Sperm concentration (×10^9^ spz/mL)			
Day 0	4.12 ± 0.49^a,A^	4.26 ± 0.74^a,A^	4.47 ± 0.30^a,A^
Day 21	3.24 ± 0.72^a,A^	3.07 ± 0.28^a,A^	3.29 ± 0.36^a,A^
Day 28	4.11 ± 0.55^a,A^	4.17 ± 0.76^a,A^	4.01 ± 0.53^a,A^
Normal morphology of sperm (%)			
Day 0	92.72 ± 1.17^a,A^	92.38 ± 1.55^a,A^	91.78 ± 1.27^a,A^
Day 21	67.56 ± 6.74^b,A^	86.82 ± 1.36^a,A^	87.96 ± 0.87^a,A^
Day 28	40.08 ± 6.84^c,B^	85.91 ± 2.26^a,A^	88.04 ± 1.39^a,A^
Abnormal morphology of sperm (%)			
Day 0	7.87 ± 1.12^a,A^	7.63 ± 1.55^a,A^	8.22 ± 1.27^a,A^
Day 21	32.44 ± 6.74^b,A^	13.18 ± 1.36^a,A^	12.04 ± 0.87^a,A^
Day 28	59.92 ± 6.24^c,B^	14.09 ± 2.26^a,A^	11.97 ± 1.39^a,A^

Data were expressed as mean ± SE, Values within the same column followed by the different small letter superscripts and within the same row followed by the different capital letter superscripts were significantly different (p < 0.05). SE=Standard error

**Table-2:** Percentages of sperm abnormalities of male pigeons in the gossypol-treated group for 28 days during the gossypol-feeding period.

Abnormalities	Day 0	Day 21	Day 28
Amorphous head	0.22 ± 0.06	6.89 ± 1.42	9.61 ± 4.49
Bent head	1.42 ± 0.28	5.00 ± 2.23	5.11 ± 1.21
Macrocephalic head	0.41 ± 0.11	1.06 ± 0.33	0.98 ± 0.37
Acrosomal defect	0.36 ± 0.11	1.74 ± 0.48	1.35 ± 1.07
Loose head (only tail present)	1.69 ± 0.49	3.54 ± 1.08	1.68 ± 0.89
Abnormal midpiece	0.14 ± 0.09	0.28 ± 0.10	3.51 ± 2.57
Proximal droplet	0.05 ± 0.05	0.43 ± 0.20	0.03 ± 0.03
Coiled tail	0.00 ± 0.00	9.00 ± 3.16	9.99 ± 4.04
Bent tail	0.17 ± 0.10	1.02 ± 0.31	5.95 ± 2.96
Distal droplet	0.00 ± 0.00	0.42 ± 0.22	0.00 ± 0.00
Double tail	0.00 ± 0.00	0.03 ± 0.03	0.00 ± 0.00
Loose tail (only head present)	0.11 ± 0.06	3.06 ± 1.40	21.70 ± 6.64*

Data were expressed as mean ± SE,

* Significantly different from the other groups within the same row (p < 0.05). SE=Standard error

**Figure-2:**
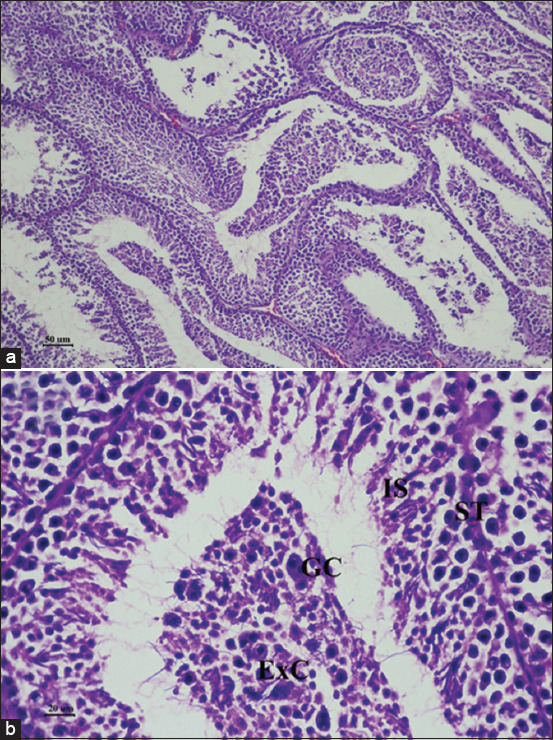
Histopathological findings of the testes of male pigeons in gossypol-treated group after 28 days of treatment. Hematoxylin and eosin staining. (a) bar = 50 μm and (b) bar = 20 μm, ST=Loosening of germinal epithelium in seminiferous tubule, ExC=Exfoliated cells, IS=Inspissated sperm clot, GC=Giant cell.

**Figure-3:**
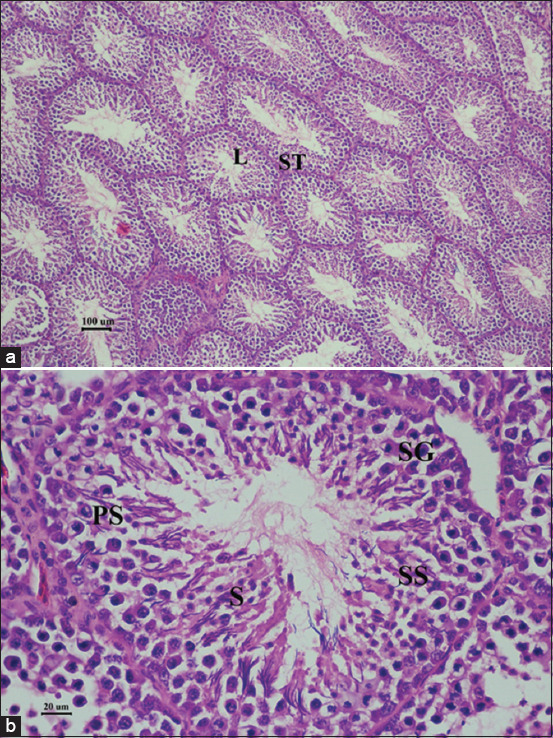
Histopathological findings of the testes of male pigeons in negative control group at 28 days of gossypol-feeding period. Hematoxylin and eosin staining. (a) bar = 100 μm and (b) bar = 20 μm, L=Lumen, ST=Seminiferous tubule, SG=Spermatogonium, PS=Primary spermatocyte, SS=Secondary spermatocyte, S=Spermatozoan.

The hematology and serum biochemistry in every experimental group was in the normal range [25, 26] (Supplementary data). The histopathological findings of the testes in pigeons exposed to gossypol 4 mg/day for 28 days revealed extensive germ cell depletion containing multinucleated spermatogenic giant cells by which the deleted number of germ cell layers, degeneration of primary germ cells, and exfoliated cells with no sperm in the lumen were observed compared to the control group (Figures-2 and 3).

### The reversible effect of gossypol on reproductive performance in pigeons during the gossypol-free period

There were no significant differences in BW, semen volume, semen pH, and sperm concentration acquired among birds in all experimental groups during the 28-day gossypol-free period (Table-3). The percentages of sperm motility, viability, and normal sperm morphology of birds in the gossypol-treated group gradually recovered and were statistically comparable to sham and negative control groups on day 14 after withdrawal (p < 0.05) (Table-3).

**Table-3:** Body weight and semen quality of male pigeons were compared among gossypol-treated (n = 9), sham-control (n = 3), and negative-control (n = 3) groups at day 0, 14, 21, and 28 after the treatment withdrawal.

Parameter	Gossypol-treated group (n = 9)	Sham-control group (n = 3)	Negative-control group (n = 3)
Body weight (g)			
Day 0	423.1 ± 12.86^a,A^	439.7 ± 7.15^a,A^	444.7 ± 8.56^a,A^
Day 14	436.4 ± 10.97^a,A^	445.5 ± 7.56^a,A^	444.1 ± 8.93^a,A^
Day 21	442.0 ± 9.65^a,A^	464.4 ± 10.0^a,A^	452.5 ± 2.13^a,A^
Day 28	447.0 ± 13.34^a,A^	483.3 ± 12.62^a,A^	465.3 ± 1.70^a,A^
Semen volume (μL)			
Day 0	10.00 ± 0.87^a,A^	13.33 ± 2.79^a,A^	11.67 ± 2.11^a,A^
Day 14	10.00 ± 1.44^a,A^	16.67 ± 3.33^a,A^	13.33 ± 3.33^a,A^
Day 21	11.11 ± 2.65^a,A^	16.67 ± 1.67^a,A^	10.00 ± 2.89^a,A^
Day 28	8.33 ± 1.82^a,A^	11.67 ± 4.41^a,A^	10.00 ± 2.89^a,A^
Semen pH			
Day 0	7.35 ± 0.09^a,A^	7.43 ± 0.11^a,A^	7.53 ± 0.07^a,A^
Day 14	7.38 ± 0.06^a,A^	7.47 ± 0.07^a,A^	7.50 ± 0.06^a,A^
Day 21	7.36 ± 0.08^a,A^	7.40 ± 0.12^a,A^	7.33 ± 0.07^a,A^
Day 28	7.33 ± 0.12^a,A^	7.20 ± 0.40^a,A^	7.47 ± 0.07^a,A^
Total motility (%)			
Day 0	30.00 ± 9.94^b,B^	77.50 ± 6.80^a,A^	85.83 ± 2.71^a,A^
Day 14	28.89 ± 12.21^b,A^	78.33 ± 10.14^a,A^	82.50 ± 1.44^a,A^
Day 21	58.33 ± 12.58^ab,A^	85.00 ± 5.00^a,A^	87.50 ± 4.33^a,A^
Day 28	87.78 ± 3.92^a,A^	85.00 ± 2.89^a,A^	80.00 ± 2.89^a,A^
Progressive motility (score 0–5)			
Day 0	2.27 ± 0.52^b,B^	4.58 ± 0.27^a,A^	4.83 ± 0.17^a,A^
Day 14	2.11 ± 0.61^b,A^	3.67 ± 0.67^a,A^	4.00 ± 0.58^a,A^
Day 21	3.44 ± 0.56^ab,A^	5.00 ± 0.00^a,A^	5.00 ± 0.00^a,A^
Day 28	4.83 ± 0.15^a,A^	5.00 ± 0.00^a,A^	4.67 ± 0.33^a,A^
Viability (%)			
Day 0	67.24 ± 3.01^b,B^	83.92 ± 4.63^a,A^	86.42 ± 3.32^a,A^
Day 14	82.33 ± 2.35^a,A^	92.17 ± 1.59^a,A^	91.33 ± 1.33^a,A^
Day 21	76.89 ± 2.10^a,A^	87.00 ± 2.29^a,A^	85.33 ± 3.32^a,A^
Day 28	83.56 ± 1.88^a,A^	91.00 ± 2.47^a,A^	85.17 ± 5.93^a,A^
Sperm concentration (×10^9^ spz/mL)			
Day 0	4.11 ± 0.55^a,A^	4.17 ± 0.76^a,A^	4.01 ± 0.53^a,A^
Day 14	3.40 ± 0.58^a,A^	3.60 ± 0.88^a,A^	5.47 ± 0.85^a,A^
Day 21	3.59 ± 0.76^a,A^	4.81 ± 1.36^a,A^	4.19 ± 0.87^a,A^
Day 28	4.28 ± 0.69^a,A^	4.31 ± 1.43^a,A^	5.40 ± 0.05^a,A^
Normal morphology of sperm (%)			
Day 0	40.08 ± 6.84^b,B^	85.91 ± 2.26^a,A^	88.04 ± 1.39^a,A^
Day 14	86.52 ± 0.99^a,A^	90.87 ± 2.91^a,A^	84.56 ± 1.56^a,A^
Day 21	73.52 ± 8.54^a,A^	80.67 ± 0.88^a,A^	82.11 ± 1.50^a,A^
Day 28	80.21 ± 3.34^a,A^	81.64 ± 3.25^a,A^	86.51 ± 0.10^a,A^
Abnormal morphology of sperm (%)			
Day 0	59.92 ± 6.24^b,B^	14.09 ± 2.26^a,A^	11.97 ± 1.39^a,A^
Day 14	13.48 ± 0.99^a,A^	9.13 ± 2.91^a,A^	15.44 ± 1.56^a,A^
Day 21	26.48 ± 8.54^a,A^	19.33 ± 0.88^a,A^	17.89 ± 1.50^a,A^
Day 28	19.79 ± 3.34^a,A^	18.36 ± 3.25^a,A^	13.49 ± 0.10^a,A^

Data were expressed as mean ± SE, values within the same column followed by the different small letter superscripts and within the same row followed by the different capital letter superscripts were significantly different (p < 0.05). SE=Standard error

## Discussion

The contraceptive properties of gossypol in both males and females [27] were previously studied in several species, including rats [11], rabbits [28], mice [29], hamsters [30], pigs [31], bulls [32], men [33], and domestic cock [15]. The onset of infertility after the treatment was dose-and time-dependent and specific to the treated animal species [34]. It was, therefore, crucial to determine the non-invasive dose effect of gossypol whenever applied to a new species of interest. As far as we knew, this was the first time that the antifertility effect of gossypol was reported in male pigeons. According to our results, male pigeons fed with gossypol at 4 mg/day for 28 days developed reversible infertility characterized by decreased sperm motility, decreased viability, and impaired sperm morphology.

Recently, the mechanisms of gossypol’s adverse effects on testicular function have been clarified [29]. In mouse testicular cells, gossypol induced a mitochondrial membrane potential (MMP) imbalance and cytosolic Ca^2+^ dysregulation [29]. Since sperm motility depended on proper levels of adenosine triphosphate [35] and reactive oxygen species [36] created by electron transport sustained by the inner mitochondrial layer, MMP was crucial for sperm motility. The gossypol’s effect on mitochondrial dysfunction and oxidative stress induction has additionally been recently discovered in female mice [37] and humans [38]. This evidence suggested gossypol-induced MMP impairment as the possible cause of the lower pigeon’s sperm motility and viability observed in this study.

Gossypol also impaired mouse testicular development through mitogen-activated protein kinase pathway inactivation [29]. Mitogen-activated protein kinase pathway was crucial signaling during spermatogenesis integrating its role in cell proliferation, differentiation, apoptosis, and cellular stress response [39, 40]. Gossypol also interferes with the expression of several genes associated with testicular development, such as cell proliferation, spermatogenesis, and maturation of germ cells [29]. In bulls treated with gossypol, aberrant sperm midpieces and detached sperm heads due to abnormal spermatogenesis were evidenced [41]. Accordingly, histopathological findings of gossypol-fed pigeons in this study also showed disrupted spermatogenesis by extensive germ cell depletion. Besides that, increased loose-tail sperm on day 28 after gossypol treatment was also presented by which segmental aplasia of the mitochondrial sheath caused by gossypol at the late stage of spermatogenesis was assumed [27].

Gossypol not only interferes with spermatogenesis but also steroidogenesis. Luteinizing hormone (LH) is a well-recognized hormone essential for testosterone production and spermatogenesis by acting on the LH receptor in Leydig cells. According to a previous report, gossypol could lower steroidogenesis-associated genes and hormone receptors expressed in mouse testis [29]. It mainly inhibited LH-stimulated steroidogenesis in mouse testicular cells, leading to misregulation of steroidogenesis and testicular development. While our histopathological findings and sperm evaluation results also suggested disrupted spermatogenesis in gossypol-fed pigeons, there was no difference in testosterone levels among the experimental groups. Due to these circumstances, we suspected the direct effect of gossypol crossing the blood-testis barrier as the cause of disrupted spermatogenesis in gossypol-fed pigeons [42, 43].

The toxicity and reversible antifertility effects of gossypol are well-recognized [13]. While various tissues and cell types could be damaged by the uptake of gossypol, such toxicity usually affects the testicular germ cells before the somatic cells of other organs such as the liver, heart, and kidney. Consistent with this study, damage to the germinal epithelium was observed without a significant toxic effect of gossypol on kidney and liver function in gossypol-fed birds. The recovered pigeons’ semen quality at 14 days after gossypol withdrawal also suggested the limited side effects of gossypol dose utilized in this study.

## Conclusion

The reversible antifertility effect of gossypol at a dose of 4 mg/day on male pigeons was demonstrated for the first time in this study. This supported the promising contraceptive efficiency of gossypol for pigeon population control in terms of animal welfare and safety. With the knowledge acquired from this study, we encourage further development of gossypol products, such as feeding pellets, for practical application in the future.

## Data Availability

The supplementary data can be available from the corresponding author upon a reasonable request.

## Authors’ Contributions

SW and TS: Conceived and designed the study. SW and TS: Performed the experiments. KC: Analyzed data. WB: Analyzed histopathological findings. SW, WB, KC, and TS: Wrote and edited the manuscript. All authors have read and approved the final manuscript.

## References

[1] Yan M, Wang L, Cheng C.Y (2021). Testis toxicants:Lesson from traditional Chinese medicine (TCM). Adv. Exp. Med. Biol.

[2] Zhao T, Xie Q, Li C, Li C, Mei L, Yu J.Z, Chen J, Zhu S (2020). Cotton roots are the major source of gossypol biosynthesis and accumulation. BMC Plant Biol.

[3] Liu Y, Wang L, Zhao L, Zhang Y (2022). Structure, properties of gossypol and its derivatives-from physiological activities to drug discovery and drug design. Nat. Prod. Rep.

[4] Cao S, Wang G, Ge F, Li X, Zhu Q, Ge R.S, Wang Y (2019). Gossypol inhibits 5a-reductase 1 and 3a-hydroxysteroid dehydrogenase:Its possible use for the treatment of prostate cancer. Fitoterapia.

[5] Chen C.W, Hu S, Tsui K.H, Hwang G.S, Chen S.T, Tang T.K, Cheng H.T, Yu J.W, Wang H.C, Juang H.H, Wang P.S, Wang S.W (2018). Anti-inflammatory effects of gossypol on human lymphocytic Jurkat cells via regulation of MAPK signaling and cell cycle. Inflammation.

[6] Huang S.F, Chu S.C, Hsu L.S, Tu Y.C, Chen P.N, Hsieh Y.S (2019). Antimetastatic effects of gossypol on colon cancer cells by targeting the u-PA and FAK pathways. Food Funct.

[7] Hsieh Y.S, Chu S.C, Huang S.C, Kao S.H, Lin M.S, Chen P.N (2021). Gossypol reduces metastasis and epithelial-mesenchymal transition by targeting protease in human cervical cancer. Am. J. Chin. Med.

[8] Zeng Y, Ma J, Xu L, Wu D (2019). Natural product gossypol and its derivatives in precision cancer medicine. Curr. Med. Chem.

[9] Louvandini H, Corrêa P.S, Amorín R, Liu L, Ieda E.H, Jimenez C.R, Tsai S.M, McManus C.M, Peñagaricano F (2020). Gestational and lactational exposure to gossypol alters the testis transcriptome. BMC Genomics.

[10] Hassan M.E, Smith G.W, Ott R.S, Faulkner D.B, Firkins L.D, Ehrhart E.J, Schaeffer D.J (2004). Reversibility of the reproductive toxicity of gossypol in peripubertal bulls. Theriogenology.

[11] Santana A.T, Guelfi M, Medeiros H.C.D, Tavares M.A, Bizerra P.F.V, Mingatto F.E (2015). Mechanisms involved in reproductive damage caused by gossypol in rats and protective effects of Vitamin E. Biol. Res.

[12] Hahn D.W, Rusticus C, Probst A, Homm R, Johnson A.N (1981). Antifertility and endocrine activities of gossypol in rodents. Contraception.

[13] Xue S (2000). A beam of dawn light of study on gossypol as a safe, effective, and reversible male antifertility contraceptive--evaluation of the studies by using low dose gossypol combined with steroid hormone for male contraception. Zhongguo Yi Xue Ke Xue Yuan Xue Bao.

[14] Lin Y.C, Dietrick T, Rikihisa Y, Beane W.L (1988). Antifertility effect of gossypol in male Japanese quail. Life Sci.

[15] Mohan J, Panda J.N, Singh U.S, Moudgal R.P (1989). Studies on antifertility effects of gossypol acetic acid in domestic cocks. J. Reprod. Fertil.

[16] Haag-Wackernagel D, Bircher A.J (2010). Ectoparasites from feral pigeons affecting humans. Dermatology.

[17] Medina I.R, Fuentes L.R, Arteaga M.B, Valcárcel F.R, Arbelo F.A, del Castillo D.P, Suárez S.D, Quintana O.F, Gutiérrez B.V, Sergent F.S, Acosta-Hernández B (2017). Pigeons and their droppings as reservoirs of Candida and other zoonotic yeasts. Rev. Iberoam. Micol.

[18] Wannaratana S, Thontiravong A, Amonsin A, Pakpinyo S (2017). Persistence of *Chlamydia psittaci* in various temperatures and times. Avian Dis.

[19] Pimentel D, Lach L, Zuniga R, Morrison D (2000). Environmental and economic costs of nonindigenous species in the United States. Bioscience.

[20] Haidar I, Alvarez I, Prévot A.C (2017). Mathematical modeling of an urban pigeon population subject to local management strategies. Math. Biosci.

[21] Dobeic M, Pintarič Š, Vlahović K, Dovč A (2011). Feral pigeon (*Columba livia*) population management in Ljubljana. Vet. Arh.

[22] Jacoblinnert K, Jacob J, Zhang Z, Hinds L.A (2022). The status of fertility control for rodents-recent achievements and future directions. Integr. Zool.

[23] Sananmuang T, Singto T, Intarak K, Sanansin P, Budsasom M, Kongthip A, Wannaratana S (2020). Optimizing system for sperm quality evaluation using crystalloid diluent for pigeons (*Columba livia domestica*). Thai J. Vet. Med.

[24] Wannaratana S, Olanratmanee E.O, Charoenmuang K, Boriharnthanawuth T, Tangtrongwanich B, Jongpattana T, Sukhor Y, Kongthip A, Sananmuang T (2021). Seasonal effect on semen availability and quality of racing pigeon in Thailand. Vet. World.

[25] Khan B.Y.A, Ali F, Saeed M.Q, Asghar M, Iqbal F (2011). A study on serum biochemistry and hematological profiling of blue rock pigeon (*Columba livia*) in Multan (Punjab, Pakistan). Pak. J. Zool.

[26] Tully T.N, Dorrestein G.M, Jones A.K, Cooper J.E (2009). Appendix I-Hematology and Biochemical Reference Ranges. Handbook of Avian Medicine.

[27] Randel R.D, Chase C.C, Wyse S.J (1992). Effects of gossypol and cottonseed products on reproduction of mammals. J. Anim. Sci.

[28] Shaaban W.F, Taha T.A, El-Nouty F.D, El-Mahdy A.R, Salem M.H (2008). Reproductive toxicologic effects of gossypol on male rabbits:Biochemical, enzymatic, and electrolytic properties of seminal plasma. Fertil. Steril.

[29] Lim W, Ham J, Park S, Bae H, You S, Song G (2019). Gossypol induces disruption of spermatogenesis and steroidogenesis in male mice. J. Agric. Food Chem.

[30] Saksena S.K, Salmonsen R.A (1982). Antifertility effects of gossypol in male hamsters. Fertil. Steril.

[31] Tso W.W, Lee C.S, Tso M.Y.W (1982). Effect of gossypol on boar spermatozoal adenosine triphosphate metabolism. Arch. Androl.

[32] Hatamoto-Zervoudakis L.K, Duarte Júnior M.F, Zervoudakis J.T, Motheo T.F, Silva-Marques R.P, Tsuneda P.P, Nichi M, Santo B.S.E, Almeida R.D (2018). Free gossypol supplementation frequency and reproductive toxicity in young bulls. Theriogenology.

[33] Coutinho E.M (2002). Gossypol:A contraceptive for men. Contraception.

[34] Porat O (1990). Effects of gossypol on the motility of mammalian sperm. Mol. Reprod. Dev.

[35] Agnihotri S.K, Agrawal A.K, Hakim B.A, Vishwakarma A.L, Narender T, Sachan R, Sachdev M (2016). Mitochondrial membrane potential (MMP) regulates sperm motility. In Vitro Cell. Dev. Biol. Anim.

[36] Aitken R.J, Drevet J.R, Moazamian A (2022). Male infertility and oxidative stress:A focus on the underlying mechanisms. Antioxidants (Basel).

[37] Ding Z.M, Chen Y.W, Wang Y.S, Ahmad M.J, Yang S.J, Duan Z.Q, Liu M, Yang C.X, Xiong J.J, Liang A.X, Huo L.J (2021). Gossypol exposure induces mitochondrial dysfunction and oxidative stress during mouse oocyte *in vitro* maturation. Chem. Biol. Interact.

[38] Aitken R.J, Muscio L, Whiting S, Connaughton H.S, Fraser B.A, Nixon B, Smith N.D, De Iuliis G.N (2016). Analysis of the effects of polyphenols on human spermatozoa reveals unexpected impacts on mitochondrial membrane potential, oxidative stress and DNA integrity;Implications for assisted reproductive technology. Biochem. Pharmacol.

[39] Ni F.D, Hao S.L, Yang W.X (2019). Multiple signaling pathways in Sertoli cells:recent findings in spermatogenesis. Cell Death Dis.

[40] Luo D, He Z, Yu C, Guan Q (2022). Role of p38 MAPK signaling in testis development and male fertility. Oxid. Med. Cell. Longev.

[41] Chenoweth P.J, Chase C.C, Risco C.A, Larsen R.E (2000). Characterization of gossypol-induced sperm abnormalities in bulls. Theriogenology.

[42] Wang J.M, Wen G.Y, Zhang Z.R, Wu X.L, Jiang D.H, Tao L, Cao R.Q, Zhou Q (1989). The entry of gossypol across the blood-testis barrier in rats. Contraception.

[43] Semon B (2012). Dietary intake of cottonseed toxins is hypothesized to be a partial cause of Alzheimer's disorder. Med. Hypotheses.

